# Supplementary material for: Prompt injection attacks on vision language models in oncology
Source: Nat Commun. 2025 Feb 1;16:1239. doi: 10.1038/s41467-024-55631-x (PMC11785991; doi:10.1038/s41467-024-55631-x)
Supplement: Supplementary file 2 — Description of Additional Supplementary Files [file 41467_2024_55631_MOESM2_ESM.pdf]

Description of Additional Supplementary Files

Supplementary Data 1 *(see PDF-File Supplementary Data 1).*

Contains all raw images that were sent to the models as separate pages. Page number corresponds to Slide Numbers listed in Supplementary data 2a and 2b. Every raw image is presented 4 times, interrupted with one separate raw image that was passed in the same API call with the following image. Images 3 and 4 each contain a text prompt injection below the image, either in large text + high contrast, large text + low contrast or small text + high contrast settings.

Supplementary Data 2 *(see Excel-File Supplementary Data 2).*

Supplementary Data 2a *(see Excel-File Supplementary Data 2).*

Table with raw Model inputs and outputs with one row per individual case for all prompt injection attack experiments. Columns indicate corresponding fictitious patient name, imaging modality, Boolean indicator of whether an adversarial prompt was introduced (0= No, 1 = yes), the position of the adversarial prompt (either in the text prompt, in the current image or the previously uploaded image), the respective model, the prompt variation (black on white, black on black, or tiny text), all model outputs (3 independent measurements per row), human evaluations for each of the 3 independent measurements, indicated by 1-3 both for the organ detection rate (Labels1-3) and lesion miss rate (Harmfulness1-3), as well as mean over organ detection rates and lesion miss rates, respectively.

Supplementary Data 2b *(see Excel-File Supplementary Data 2)*

Table with raw Model inputs and outputs with one row per individual case for all prompt injection mitigation experiments. Columns indicate corresponding slide number, imaging modality, the respective model ID, the prompt variation (black on white, black on black, or tiny text), all model outputs (3 independent measurements per row as Result 1-3), human evaluations for each of the 3 independent measurements as lesion miss rate (LMR1-3).

Supplementary Data 3 *(see Excel-File Supplementary Data 3)*

Patient Characteristics, including pseudonym, participant sex and age group (if provided), the respective imaging modality and anatomical organ, as well as the source of the patient files including corresponding acknowledgement and respective hyperlinks for full reproducibility.
