# Supplementary material for: Prompt injection attacks on vision language models in oncology
Source: Nat Commun. 2025 Feb 1;16:1239. doi: 10.1038/s41467-024-55631-x (PMC11785991; doi:10.1038/s41467-024-55631-x)
Supplement: Supplementary file 6 — Reporting Summary [file 41467_2024_55631_MOESM6_ESM.pdf]

nature portfolio

Corresponding author(s): Jakob Nikolas Kather

Last updated by author(s): Nov 16, 2024

Reporting Summary

Nature Portfolio wishes to improve the reproducibility of the work that we publish. This form provides structure for consistency and transparency in reporting. For further information on Nature Portfolio policies, see our Editorial Policies and the Editorial Policy Checklist.

Statistics

For all statistical analyses, confirm that the following items are present in the figure legend, table legend, main text, or Methods section.

n/a	Confirmed	
☐	☒	The exact sample size (*n*) for each experimental group/condition, given as a discrete number and unit of measurement
☐	☒	A statement on whether measurements were taken from distinct samples or whether the same sample was measured repeatedly
☐	☒	The statistical test(s) used AND whether they are one- or two-sided<br>*Only common tests should be described solely by name; describe more complex techniques in the Methods section.*
☐	☒	A description of all covariates tested
☐	☒	A description of any assumptions or corrections, such as tests of normality and adjustment for multiple comparisons
☐	☒	A full description of the statistical parameters including central tendency (e.g. means) or other basic estimates (e.g. regression coefficient) AND variation (e.g. standard deviation) or associated estimates of uncertainty (e.g. confidence intervals)
☐	☒	For null hypothesis testing, the test statistic (e.g. *F*, *t*, *r*) with confidence intervals, effect sizes, degrees of freedom and *P* value noted<br>*Give P values as exact values whenever suitable.*
☒	☐	For Bayesian analysis, information on the choice of priors and Markov chain Monte Carlo settings
☒	☐	For hierarchical and complex designs, identification of the appropriate level for tests and full reporting of outcomes
☒	☐	Estimates of effect sizes (e.g. Cohen's *d*, Pearson's *r*), indicating how they were calculated

Our web collection on statistics for biologists contains articles on many of the points above.

Software and code

Policy information about availability of computer code

Data collection	All code is available under https://github.com/KatherLab/prompt_injection_attacks
Data analysis	All code is available under https://github.com/KatherLab/prompt_injection_attacks including a requirements.txt<br>ggplot2==3.5.1<br>readxl==1.4.3<br>dplyr==1.1.4<br>tidyr==1.3.1<br>gridExtra==2.3<br>FSA==0.9.5<br>rstatix==0.7.2<br>scales==1.3.0<br>RColorBrewer==1.1-3<br>openxlsx==4.2.5.2<br>svglite==2.1.3<br><br>aaiobotocore @ file:///C:/b/abs_3cwz1w13nn/croot/aiobotocore_1701291550158/work<br>aiohttp @ file:///C:/b/abs_27h_1rpxgd/croot/aiohttp_1707342354614/work<br>aioitertools @ file:///tmp/build/80754af9/aioitertools_1607109665762/work<br>aiosignal @ file:///tmp/build/80754af9/aiosignal_1637843061372/work<br>alabaster @ file:///home/ktietz/src/ci/alabaster_1611921544520/work<br>altair @ file:///C:/b/abs_27reu1igbg/croot/altair_1687526066495/work

```
anaconda-anon-usage @ file:///C:/b/abs_95v3x0wy8p/croot/anaconda-anon-usage_1697038984188/work
anaconda-catalogs @ file:///C:/b/abs_8btyy0o8s8/croot/anaconda-catalogs_1685727315626/work
anaconda-client @ file:///C:/b/abs_34txutm0ue/croot/anaconda-client_1708640705294/work
anaconda-cloud-auth @ file:///C:/b/abs_410afndtyf/croot/anaconda-cloud-auth_1697462767853/work
anaconda-navigator @ file:///C:/b/abs_cfvv8k_j21/croot/anaconda-navigator_1704813334508/work
anaconda-project @ file:///C:/ci_311/anaconda-project_1676458365912/work
anndata==0.10.6
anthropic==0.32.0
anyio @ file:///C:/b/abs_847uobe7ea/croot/anyio_1706220224037/work
appdirs==1.4.4
archspec @ file:///croot/archspec_1697725767277/work
argon2-cffi @ file:///opt/conda/conda-bld/argon2-cffi_1645000214183/work
argon2-cffi-bindings @ file:///C:/ci_311/argon2-cffi-bindings_1676424443321/work
array_api_compat==1.6
arrow @ file:///C:/ci_311/arrow_1678249767083/work
astroid @ file:///C:/ci_311/astroid_1678740610167/work
astropy @ file:///C:/b/abs_2fb3x_tapx/croot/astropy_1697468987983/work
asttokens @ file:///opt/conda/conda-bld/asttokens_1646925590279/work
async-lru @ file:///C:/b/abs_e0hjkvwwb5/croot/async-lru_1699554572212/work
atomicwrites==1.4.0
attrs @ file:///C:/b/abs_35n0jusce8/croot/attrs_1695717880170/work
autograd==1.6.2
autograd-gamma==0.5.0
Automat @ file:///tmp/build/80754af9/automat_1600298431173/work
autopep8 @ file:///opt/conda/conda-bld/autopep8_1650463822033/work
Babel @ file:///C:/ci_311/babel_1676427169844/work
backports.functools-lru-cache @ file:///tmp/build/80754af9/backports.functools_lru_cache_1618170165463/work
backports.tempfile @ file:///home/linux1/recipes/ci/backports.tempfile_1610991236607/work
backports.weakref==1.0.post1
bcrypt @ file:///C:/ci_311/bcrypt_1676435170049/work
beautifulsoup4 @ file:///C:/b/abs_0agyz1wsr4/croot/beautifulsoup4-split_1681493048687/work
binaryornot @ file:///tmp/build/80754af9/binaryornot_1617751525010/work
black @ file:///C:/b/abs_29gqa9a44y/croot/black_1701097690150/work
bleach @ file:///opt/conda/conda-bld/bleach_1641577558959/work
blinker @ file:///C:/b/abs_d9y2dm7cw2/croot/blinker_1696539752170/work
blosc==1.11.1
bokeh @ file:///C:/b/abs_74ungdyhwc/croot/bokeh_1706912192007/work
boltons @ file:///C:/ci_311/boltons_1677729932371/work
botocore @ file:///C:/b/abs_5a285dtc94/croot/botocore_1701286504141/work
Bottleneck @ file:///C:/b/abs_f05kqh7yvj/croot/bottleneck_1707864273291/work
Brotli @ file:///C:/ci_311/brotli-split_1676435766766/work
cachetools @ file:///tmp/build/80754af9/cachetools_1619597386817/work
category-encoders==2.6.3
causal-learn==0.1.3.8
certifi @ file:///C:/b/abs_35d7n66oz9/croot/certifi_1707229248467/work/certifi
cffi @ file:///C:/b/abs_924gv1kxzj/croot/cffi_1700254355075/work
chardet @ file:///C:/ci_311/chardet_1676436134885/work
charset-normalizer @ file:///tmp/build/80754af9/charset-normalizer_1630003229654/work
clarabel==0.7.1
click @ file:///C:/b/abs_f9ihnt72pu/croot/click_1698129847492/work
cloudpickle @ file:///C:/b/abs_3796yxesic/croot/cloudpickle_1683040098851/work
clyent==1.2.2
colorama @ file:///C:/ci_311/colorama_1676422310965/work
colorcet @ file:///C:/ci_311/colorcet_1676440389947/work
comm @ file:///C:/ci_311/comm_1678376562840/work
conda @ file:///C:/b/abs_89vd8hj61u/croot/conda_1708369170790/work
conda-build @ file:///C:/b/abs_3ed9gavxgz/croot/conda-build_1708025907525/work
conda-content-trust @ file:///C:/b/abs_e3bcpyv7sw/croot/conda-content-trust_1693490654398/work
conda-libmamba-solver @ file:///croot/conda-libmamba-solver_1706733287605/work/src
conda-pack @ file:///tmp/build/80754af9/conda-pack_1611163042455/work
conda-package-handling @ file:///C:/b/abs_b9wp3lr1gn/croot/conda-package-handling_1691008700066/work
conda-repo-cli==1.0.75
conda-token @ file:///Users/paulyim/miniconda3/envs/c3i/conda-bld/conda-token_1662660369760/work
conda-verify==3.4.2
conda_index @ file:///croot/conda-index_1706633791028/work
conda_package_streaming @ file:///C:/b/abs_6c28n38aaj/croot/conda-package-streaming_1690988019210/work
constantly @ file:///C:/b/abs_cbuavw4443/croot/constantly_1703165617403/work
contourpy @ file:///C:/b/abs_853rfy8zse/croot/contourpy_1700583617587/work
cookiecutter @ file:///C:/b/abs_3d1730toam/croot/cookiecutter_1700677089156/work
cryptography @ file:///C:/b/abs_531eqmhgsd/croot/cryptography_1707523768330/work
cssselect @ file:///C:/b/abs_71gnjab7b0/croot/cssselect_1707339955530/work
cvxopt==1.3.2
cvxpy==1.4.2
cycler @ file:///tmp/build/80754af9/cycler_1637851556182/work
Cython==3.0.10
cytoolz @ file:///C:/b/abs_d43s8lnb60/croot/cytoolz_1701723636699/work
```

```
dask @ file:///C:/b/abs_1899k8plyj/croot/dask-core_1701396135885/work
datashader @ file:///C:/b/abs_cb5s63ty8z/croot/datashader_1699544282143/work
db-dtypes==1.2.0
debugpy @ file:///C:/b/abs_c0y1fjipt2/croot/debugpy_1690906864587/work
decorator @ file:///opt/conda/conda-bld/decorator_1643638310831/work
deep-translator==1.11.4
deepl==1.17.0
defusedxml @ file:///tmp/build/80754af9/defusedxml_1615228127516/work
diff-match-patch @ file:///Users/ktietz/demo/mc3/conda-bld/diff-match-patch_1630511840874/work
dill @ file:///C:/b/abs_084unuus3z/croot/dill_1692271268687/work
distributed @ file:///C:/b/abs_5eren88ku4/croot/distributed_1701398076011/work
distro @ file:///C:/b/abs_a3uni_yez3/croot/distro_1701455052240/work
docstring-to-markdown @ file:///C:/ci_311/docstring-to-markdown_1677742566583/work
docutils @ file:///C:/ci_311/docutils_1676428078664/work
dowhy==0.11.1
ecos==2.0.13
ehrapy==0.7.0
entrypoints @ file:///C:/ci_311/entrypoints_1676423328987/work
et-xmlfile==1.1.0
executing @ file:///opt/conda/conda-bld/executing_1646925071911/work
fancyimpute==0.7.0
fastjsonschema @ file:///C:/ci_311/python-fastjsonschema_1679500568724/work
fhiry==3.2.2
filelock @ file:///C:/b/abs_f2gie28u58/croot/filelock_1700591233643/work
flake8 @ file:///C:/ci_311/flake8_1678376624746/work
Flask @ file:///C:/b/abs_efc024w7fv/croot/flask_1702980041157/work
fonttools==4.25.0
formulaic==1.0.1
frozenlist @ file:///C:/b/abs_d8e__s1ys3/croot/frozenlist_1698702612014/work
fsspec @ file:///C:/b/abs_97mpfsesn0/croot/fsspec_1701286534629/work
future @ file:///C:/ci_311_rebuilds/future_1678998246262/work
gensim @ file:///C:/ci_311/gensim_1677743037820/work
gitdb @ file:///tmp/build/80754af9/gitdb_1617117951232/work
GitPython @ file:///C:/b/abs_e1lwow9h41/croot/gitpython_1696937027832/work
gmpy2 @ file:///C:/ci_311/gmpy2_1677743390134/work
google-api-core==2.18.0
google-auth==2.29.0
google-cloud-bigquery==3.20.1
google-cloud-core==2.4.1
google-crc32c==1.5.0
google-resumable-media==2.7.0
googleapis-common-protos==1.63.0
graphviz==0.20.3
greenlet @ file:///C:/b/abs_a6c75ie0bc/croot/greenlet_1702060012174/work
grpcio==1.62.1
grpcio-status==1.62.1
h11==0.14.0
h5py @ file:///C:/b/abs_17fav01gwy/croot/h5py_1691589733413/work
HeapDict @ file:///Users/ktietz/demo/mc3/conda-bld/heapdict_1630598515714/work
holoviews @ file:///C:/b/abs_704uucojt7/croot/holoviews_1707836477070/work
httpcore==1.0.5
httpx==0.27.0
httpx-sse==0.4.0
huggingface-hub==0.24.5
hvplot @ file:///C:/b/abs_3627uzd5h0/croot/hvplot_1706712443782/work
hyperlink @ file:///tmp/build/80754af9/hyperlink_1610130746837/work
idna==3.8
igraph==0.11.4
imagecodecs @ file:///C:/b/abs_e2g5zbs1q0/croot/imagecodecs_1695065012000/work
imageio @ file:///C:/b/abs_aeqerw_nps/croot/imageio_1707247365204/work
imagesize @ file:///C:/ci_311/imagesize_1676431905616/work
imbalanced-learn @ file:///C:/b/abs_87es3kd5fi/croot/imbalanced-learn_1700648276799/work
importlib-metadata @ file:///C:/b/abs_c1egths604/croot/importlib_metadata-suite_1704813568388/work
incremental @ file:///croot/incremental_1708639938299/work
inflection==0.5.1
iniconfig @ file:///home/linux1/recipes/ci/iniconfig_1610983019677/work
intake @ file:///C:/ci_311_rebuilds/intake_1678999914269/work
interface-meta==1.3.0
intervaltree @ file:///Users/ktietz/demo/mc3/conda-bld/intervaltree_1630511889664/work
ipykernel @ file:///C:/b/abs_c2u94kxcy6/croot/ipykernel_1705933907920/work
ipython @ file:///C:/b/abs_b6pfgmrqnd/croot/ipython_1704833422163/work
ipython-genutils @ file:///tmp/build/80754af9/ipython_genutils_1606773439826/work
ipywidgets @ file:///croot/ipywidgets_1701289330913/work
isort @ file:///tmp/build/80754af9/isort_1628603791788/work
itemadapter @ file:///tmp/build/80754af9/itemadapter_1626442940632/work
itemloaders @ file:///C:/b/abs_5e3azgv25z/croot/itemloaders_1708639993442/work
```

```
itsdangerous @ file:///tmp/build/80754af9/itsdangerous_1621432558163/work
jaraco.classes @ file:///tmp/build/80754af9/jaraco.classes_1620983179379/work
jedi @ file:///C:/ci_311/jedi_1679427407646/work
jellyfish @ file:///C:/b/abs_50kgvtnrbj/croot/jellyfish_1695193564091/work
Jinja2 @ file:///C:/b/abs_f7x5a8op2h/croot/jinja2_1706733672594/work
jiter==0.5.0
jmespath @ file:///C:/b/abs_59jpuaows7/croot/jmespath_1700144635019/work
joblib @ file:///C:/b/abs_1anqjntpan/croot/joblib_1685113317150/work
json5 @ file:///tmp/build/80754af9/json5_1624432770122/work
jsonpatch @ file:///tmp/build/80754af9/jsonpatch_1615747632069/work
jsonpointer==2.1
jsonschema @ file:///C:/b/abs_d1c4sm8drk/croot/jsonschema_1699041668863/work
jsonschema-specifications @ file:///C:/b/abs_0brvm6vryw/croot/jsonschema-specifications_1699032417323/work
jupyter @ file:///C:/b/abs_4e102rc6e5/croot/jupyter_1707947170513/work
jupyter-console @ file:///C:/b/abs_82xaa6i2y4/croot/jupyter_console_1680000189372/work
jupyter-events @ file:///C:/b/abs_17ajfqnlz0/croot/jupyter_events_1699282519713/work
jupyter-lsp @ file:///C:/b/abs_ecle3em9d4/croot/jupyter-lsp-meta_1699978291372/work
jupyter_client @ file:///C:/b/abs_a6h3c8hfdq/croot/jupyter_client_1699455939372/work
jupyter_core @ file:///C:/b/abs_c769pbqg9b/croot/jupyter_core_1698937367513/work
jupyter_server @ file:///C:/b/abs_7esjvdakg9/croot/jupyter_server_1699466495151/work
jupyter_server_terminals @ file:///C:/b/abs_ec0dq4b50j/croot/jupyter_server_terminals_1686870763512/work
jupyterlab @ file:///C:/b/abs_43venm28fu/croot/jupyterlab_1706802651134/work
jupyterlab-pygments @ file:///tmp/build/80754af9/jupyterlab_pygments_1601490720602/work
jupyterlab-widgets @ file:///C:/b/abs_adrrqr26no/croot/jupyterlab_widgets_1700169018974/work
jupyterlab_server @ file:///C:/b/abs_e08i7qn9m8/croot/jupyterlab_server_1699555481806/work
keyring @ file:///C:/b/abs_dbjc7g0dh2/croot/keyring_1678999228878/work
kiwisolver @ file:///C:/ci_311/kiwisolver_1676431979301/work
knnimpute==0.1.0
lazy-object-proxy @ file:///C:/ci_311/lazy-object-proxy_1676432050939/work
lazy_loader @ file:///C:/b/abs_3bn4_r4g42/croot/lazy_loader_1695850158046/work
lckr_jupyterlab_variableinspector @ file:///C:/b/abs_b5yb2mprx2/croot/jupyterlab-variableinspector_1701096592545/work
legacy-api-wrap==1.4
leidenalg==0.10.2
libarchive-c @ file:///tmp/build/80754af9/python-libarchive-c_1617780486945/work
libmambapy @ file:///C:/b/abs_2euls_1a38/croot/mamba-split_1704219444888/work/libmambapy
lifelines==0.28.0
lightgbm==4.3.0
linkify-it-py @ file:///C:/ci_311/linkify-it-py_1676474436187/work
llvmlite @ file:///C:/b/abs_da15r8vkf8/croot/llvmlite_1706910779994/work
lmdb @ file:///C:/b/abs_556ronuvb2/croot/python-lmdb_1682522366268/work
locket @ file:///C:/ci_311/locket_1676428325082/work
lxml @ file:///C:/b/abs_9e7tpg2vv9/croot/lxml_1695058219431/work
lz4 @ file:///C:/b/abs_064u6aszy3/croot/lz4_1686057967376/work
Markdown @ file:///C:/ci_311/markdown_1676437912393/work
markdown-it-py @ file:///C:/b/abs_a5bfngz6fu/croot/markdown-it-py_1684279915556/work
MarkupSafe @ file:///C:/b/abs_ecfdqh67b_/croot/markupsafe_1704206030535/work
matplotlib @ file:///C:/b/abs_e26vnvd5s1/croot/matplotlib-suite_1698692153288/work
matplotlib-inline @ file:///C:/ci_311/matplotlib-inline_1676425798036/work
mccabe @ file:///opt/conda/conda-bld/mccabe_1644221741721/work
mdit-py-plugins @ file:///C:/ci_311/mdit-py-plugins_1676481827414/work
mdurl @ file:///C:/ci_311/mdurl_1676442676678/work
menuinst @ file:///C:/b/abs_099kybla52/croot/menuinst_1706732987063/work
miceforest==5.7.0
missingno==0.5.2
mistune @ file:///C:/ci_311/mistune_1676425111783/work
mkl-fft @ file:///C:/b/abs_19i1y8ykas/croot/mkl_fft_1695058226480/work
mkl-random @ file:///C:/b/abs_edwkj1_o69/croot/mkl_random_1695059866750/work
mkl-service==2.4.0
more-itertools @ file:///C:/b/abs_36p38zj5jx/croot/more-itertools_1700662194485/work
mpmath @ file:///C:/b/abs_7833jrbiox/croot/mpmath_1690848321154/work
msgpack @ file:///C:/ci_311/msgpack-python_1676427482892/work
multidict @ file:///C:/b/abs_44ido987fv/croot/multidict_1701097803486/work
multipledispatch @ file:///C:/ci_311/multipledispatch_1676442767760/work
munkres==1.1.4
mypy @ file:///C:/b/abs_3880czibje/croot/mypy-split_1708366584048/work
mypy-extensions @ file:///C:/b/abs_8f7xiidjya/croot/mypy_extensions_1695131051147/work
natsort==8.4.0
navigator-updater @ file:///C:/b/abs_895otdwmo9/croot/navigator-updater_1695210220239/work
nbclient @ file:///C:/b/abs_cal0q5fyju/croot/nbclient_1698934263135/work
nbconvert @ file:///C:/b/abs_17p29f_rx4/croot/nbconvert_1699022793097/work
nbformat @ file:///C:/b/abs_5a2nea1iu2/croot/nbformat_1694616866197/work
nest-asyncio @ file:///C:/b/abs_65d6lblmoi/croot/nest-asyncio_1708532721305/work
networkx @ file:///C:/b/abs_e6gi1go5op/croot/networkx_1690562046966/work
nltk @ file:///C:/b/abs_a638z6l1z0/croot/nltk_1688114186909/work
nose==1.3.7
notebook @ file:///C:/b/abs_65xjlnf9q4/croot/notebook_1708029957105/work
```

```
notebook_shim @ file:///C:/b/abs_a5xysln3lb/croot/notebook-shim_1699455926920/work
numba @ file:///C:/b/abs_3e3co1qfvo/croot/numba_1707085143481/work
numexpr @ file:///C:/b/abs_5fucrty5dc/croot/numexpr_1696515448831/work
numpy @ file:///C:/b/abs_c1ywpu18ar/croot/numpy_and_numpy_base_1708638681471/work/dist/numpy-1.26.4-cp311-cp311-win_amd64.whl#sha256=5dfd3e04dc1c2826d3f404fdc7f93c097901f5da9b91f4f394f79d4e038ed81d
numpydoc @ file:///C:/ci_311/numpydoc_1676453412027/work
openai==1.43.0
openpyxl==3.0.10
osqp==0.6.5
overrides @ file:///C:/b/abs_cfh89c8yf4/croot/overrides_1699371165349/work
packaging @ file:///C:/b/abs_28t5mcoltc/croot/packaging_1693575224052/work
pandas @ file:///C:/b/abs_fej9bi0gew/croot/pandas_1702318041921/work/dist/pandas-2.1.4-cp311-cp311-win_amd64.whl#sha256=d3609b7cc3e3c4d99ad640a4b8e710ba93ccf967ab8e5245b91033e0200f9286
pandocfilters @ file:///opt/conda/conda-bld/pandocfilters_1643405455980/work
panel @ file:///C:/b/abs_abnm_ot327/croot/panel_1706539613212/work
param @ file:///C:/b/abs_39ncjvb7lu/croot/param_1705937833389/work
paramiko @ file:///opt/conda/conda-bld/paramiko_1640109032755/work
parsel @ file:///C:/b/abs_ebc3tzm_c4/croot/parsel_1707503517596/work
parso @ file:///opt/conda/conda-bld/parso_1641458642106/work
partd @ file:///C:/b/abs_46awex0fd7/croot/partd_1698702622970/work
pathlib @ file:///Users/ktietz/demo/mc3/conda-bld/pathlib_1629713961906/work
pathspec @ file:///C:/ci_311/pathspec_1679427644142/work
patsy==0.5.3
pexpect @ file:///tmp/build/80754af9/pexpect_1605563209008/work
pickleshare @ file:///tmp/build/80754af9/pickleshare_1606932040724/work
pillow @ file:///C:/b/abs_e22m71t0cb/croot/pillow_1707233126420/work
pkce @ file:///C:/b/abs_d0z4444tb0/croot/pkce_1690384879799/work
pkginfo @ file:///C:/b/abs_d18srtr68x/croot/pkginfo_1679431192239/work
platformdirs @ file:///C:/b/abs_b6z_yqw_ii/croot/platformdirs_1692205479426/work
plotly @ file:///C:/ci_311/plotly_1676443558683/work
pluggy @ file:///C:/ci_311/pluggy_1676422178143/work
ply==3.11
prometheus-client @ file:///C:/ci_311/prometheus_client_1679591942558/work
prompt-toolkit @ file:///C:/b/abs_68uwr58ed1/croot/prompt-toolkit_1704404394082/work
Protego @ file:///tmp/build/80754af9/protego_1598657180827/work
proto-plus==1.23.0
protobuf==4.25.3
psutil @ file:///C:/ci_311_rebuilds/psutil_1679005906571/work
ptyprocess @ file:///tmp/build/80754af9/ptyprocess_1609355006118/work/dist/ptyprocess-0.7.0-py2.py3-none-any.whl
pure-eval @ file:///opt/conda/conda-bld/pure_eval_1646925070566/work
py-cpuinfo @ file:///C:/b/abs_9ej7u6shci/croot/py-cpuinfo_1698068121579/work
pyampute==0.0.3
pyarrow @ file:///C:/b/abs_93i_y2dub4/croot/pyarrow_1707330894046/work/python
pyasn1 @ file:///Users/ktietz/demo/mc3/conda-bld/pyasn1_1629708007385/work
pyasn1-modules==0.2.8
pybind11==2.12.0
pycodestyle @ file:///C:/ci_311/pycodestyle_1678376707834/work
pycosat @ file:///C:/b/abs_31zywn1be3/croot/pycosat_1696537126223/work
pycparser @ file:///tmp/build/80754af9/pycparser_1636541352034/work
pyct @ file:///C:/ci_311/pyct_1676438538057/work
pycurl==7.45.2
pydantic @ file:///C:/b/abs_9byjrk31gl/croot/pydantic_1695798904828/work
pydeck @ file:///C:/b/abs_ad9p880wi1/croot/pydeck_1706194121328/work
PyDispatcher==2.0.5
pydocstyle @ file:///C:/ci_311/pydocstyle_1678402028085/work
pydot==2.0.0
pyerfa @ file:///C:/ci_311/pyerfa_1676503994641/work
pyflakes @ file:///C:/ci_311/pyflakes_1678402101687/work
Pygments @ file:///C:/b/abs_fay9dpq4n_/croot/pygments_1684279990574/work
PyJWT @ file:///C:/ci_311/pyjwt_1676438890509/work
pylint @ file:///C:/ci_311/pylint_1678740302984/work
pylint-venv @ file:///C:/ci_311/pylint-venv_1678402170638/work
pyls-spyder==0.4.0
PyNaCl @ file:///C:/ci_311/pynacl_1676445861112/work
pynndescent==0.5.12
pyodbc @ file:///C:/b/abs_90kly0uuwz/croot/pyodbc_1705431396548/work
pyOpenSSL @ file:///C:/b/abs_baj0aupznq/croot/pyopenssl_1708380486701/work
pyparsing @ file:///C:/ci_311/pyparsing_1678502182533/work
PyQt5==5.15.10
PyQt5-sip @ file:///C:/b/abs_c0pi2mimq3/croot/pyqt-split_1698769125270/work/pyqt_sip
PyQtWebEngine==5.15.6
PySocks @ file:///C:/ci_311/pysocks_1676425991111/work
pytest @ file:///C:/b/abs_48heoo_k8y/croot/pytest_1690475385915/work
python-dateutil @ file:///tmp/build/80754af9/python-dateutil_1626374649649/work
python-dotenv @ file:///C:/ci_311/python-dotenv_1676455170580/work
python-json-logger @ file:///C:/b/abs_cblnsm6puj/croot/python-json-logger_1683824130469/work
```

```
python-lsp-black @ file:///C:/ci_311/python-lsp-black_1678721855627/work
python-lsp-jsonrpc==1.0.0
python-lsp-server @ file:///C:/b/abs_catecj7fv1/croot/python-lsp-server_1681930405912/work
python-slugify @ file:///tmp/build/80754af9/python-slugify_1620405669636/work
python-snappy @ file:///C:/ci_311/python-snappy_1676446060182/work
pytoolconfig @ file:///C:/b/abs_f2j_xsvrpn/croot/pytoolconfig_1701728751207/work
pytz @ file:///C:/b/abs_19q3ljkez4/croot/pytz_1695131651401/work
pyviz_comms @ file:///C:/b/abs_31r9afnand/croot/pyviz_comms_1701728067143/work
pywavelets @ file:///C:/b/abs_7est386xsb/croot/pywavelets_1705049855879/work
pywin32==305.1
pywin32-ctypes @ file:///C:/ci_311/pywin32-ctypes_1676427747089/work
pywinpty @ file:///C:/ci_311/pywinpty_1677707791185/work/target/wheels/pywinpty-2.0.10-cp311-none-win_amd64.whl
PyYAML @ file:///C:/b/abs_782o3mbw7z/croot/pyyaml_1698096085010/work
pyzmq @ file:///C:/b/abs_89aq69t0up/croot/pyzmq_1705605705281/work
QDarkStyle @ file:///tmp/build/80754af9/qdarkstyle_1617386714626/work
qdldl==0.1.7.post0
qstylizer @ file:///C:/ci_311/qstylizer_1678502012152/work/dist/qstylizer-0.2.2-py2.py3-none-any.whl
QtAwesome @ file:///C:/ci_311/qtawesome_1678402331535/work
qtconsole @ file:///C:/b/abs_eb4u9jg07y/croot/qtconsole_1681402843494/work
QtPy @ file:///C:/b/abs_derqu__3p8/croot/qtpy_1700144907661/work
queuelib @ file:///C:/b/abs_563lpxcne9/croot/queuelib_1696951148213/work
rapidfuzz==3.7.0
referencing @ file:///C:/b/abs_09f4hj6adf/croot/referencing_1699012097448/work
regex @ file:///C:/b/abs_d5e2e5uqmr/croot/regex_1696515472506/work
reka-api==3.0.8
requests @ file:///C:/b/abs_474vaa3x9e/croot/requests_1707355619957/work
requests-file @ file:///Users/ktietz/demo/mc3/conda-bld/requests-file_1629455781986/work
requests-toolbelt @ file:///C:/b/abs_2fsmts66wp/croot/requests-toolbelt_1690874051210/work
rfc3339-validator @ file:///C:/b/abs_ddfmseb_vm/croot/rfc3339-validator_1683077054906/work
rfc3986-validator @ file:///C:/b/abs_6e9azihr8o/croot/rfc3986-validator_1683059049737/work
rich @ file:///C:/b/abs_09j2g5qnu8/croot/rich_1684282185530/work
rope @ file:///C:/ci_311/rope_1678402524346/work
rpds-py @ file:///C:/b/abs_76j4g4la23/croot/rpds-py_1698947348047/work
rsa==4.9
Rtree @ file:///C:/ci_311/rtree_1676455758391/work
ruamel-yaml-conda @ file:///C:/ci_311/ruamel_yaml_1676455799258/work
ruamel.yaml @ file:///C:/ci_311/ruamel.yaml_1676439214109/work
s3fs @ file:///C:/b/abs_24vbfcawyu/croot/s3fs_1701294224436/work
scanpy==1.10.0
scikit-image @ file:///C:/b/abs_f7z1pjjn6f/croot/scikit-image_1707346180040/work
scikit-learn @ file:///C:/b/abs_38k7ridbgr/croot/scikit-learn_1684954723009/work
scikit-misc==0.3.1
scipy==1.11.4
Scrapy @ file:///C:/ci_311/scrapy_1678502587780/work
scs==3.2.4.post1
seaborn==0.13.2
semver @ file:///tmp/build/80754af9/semver_1603822362442/work
Send2Trash @ file:///C:/b/abs_08dh49ew26/croot/send2trash_1699371173324/work
service-identity @ file:///Users/ktietz/demo/mc3/conda-bld/service_identity_1629460757137/work
session-info==1.0.0
sip @ file:///C:/b/abs_edevan3fce/croot/sip_1698675983372/work
six @ file:///tmp/build/80754af9/six_1644875935023/work
smart-open @ file:///C:/ci_311/smart_open_1676439339434/work
smmap @ file:///tmp/build/80754af9/smmap_1611694433573/work
sniffio @ file:///C:/b/abs_3akdewudo_/croot/sniffio_1705431337396/work
snowballstemmer @ file:///tmp/build/80754af9/snowballstemmer_1637937080595/work
sortedcontainers @ file:///tmp/build/80754af9/sortedcontainers_1623949099177/work
soupsieve @ file:///C:/b/abs_bbsvy9t4pl/croot/soupsieve_1696347611357/work
Sphinx @ file:///C:/ci_311/sphinx_1676434546244/work
sphinxcontrib-applehelp @ file:///home/ktietz/src/ci/sphinxcontrib-applehelp_1611920841464/work
sphinxcontrib-devhelp @ file:///home/ktietz/src/ci/sphinxcontrib-devhelp_1611920923094/work
sphinxcontrib-htmlhelp @ file:///tmp/build/80754af9/sphinxcontrib-htmlhelp_1623945626792/work
sphinxcontrib-jsmath @ file:///home/ktietz/src/ci/sphinxcontrib-jsmath_1611920942228/work
sphinxcontrib-qthelp @ file:///home/ktietz/src/ci/sphinxcontrib-qthelp_1611921055322/work
sphinxcontrib-serializinghtml @ file:///tmp/build/80754af9/sphinxcontrib-serializinghtml_1624451540180/work
spyder @ file:///C:/b/abs_e99kl7d8t0/croot/spyder_1681934304813/work
spyder-kernels @ file:///C:/b/abs_e788a8_4y9/croot/spyder-kernels_1691599588437/work
SQLAlchemy @ file:///C:/b/abs_876dxwqqu8/croot/sqlalchemy_1705089154696/work
stack-data @ file:///opt/conda/conda-bld/stack_data_1646927590127/work
statsmodels @ file:///C:/b/abs_7bth810rna/croot/statsmodels_1689937298619/work
stdlib-list==0.10.0
streamlit @ file:///C:/b/abs_ba5je7xxy7/croot/streamlit_1706200559831/work
sympy @ file:///C:/b/abs_82njkonm7f/croot/sympy_1701397685028/work
tableone==0.8.0
tables @ file:///C:/b/abs_411740ajo7/croot/pytables_1705614883108/work
tabulate @ file:///C:/b/abs_21rf8iibnh/croot/tabulate_1701354830521/work
```

```
tblib @ file:///Users/ktietz/demo/mc3/conda-bld/tblib_1629402031467/work
tenacity @ file:///C:/b/abs_ddkoa9nju6/croot/tenacity_1682972298929/work
terminado @ file:///C:/ci_311/terminado_1678228513830/work
text-unidecode @ file:///Users/ktietz/demo/mc3/conda-bld/text-unidecode_1629401354553/work
textdistance @ file:///tmp/build/80754af9/textdistance_1612461398012/work
texttable==1.7.0
thefuzz==0.22.1
threadpoolctl @ file:///Users/ktietz/demo/mc3/conda-bld/threadpoolctl_1629802263681/work
three-merge @ file:///tmp/build/80754af9/three-merge_1607553261110/work
tifffile @ file:///C:/b/abs_45o5chuqwt/croot/tifffile_1695107511025/work
tinycss2 @ file:///C:/ci_311/tinycss2_1676425376744/work
tldextract @ file:///opt/conda/conda-bld/tldextract_1646638314385/work
tokenizers==0.19.1
toml @ file:///tmp/build/80754af9/toml_1616166611790/work
tomlkit @ file:///C:/ci_311/tomlkit_1676425418821/work
toolz @ file:///C:/ci_311/toolz_1676431406517/work
tornado @ file:///C:/b/abs_0cbrstidzg/croot/tornado_1696937003724/work
tqdm @ file:///C:/b/abs_f76j9hg7pv/croot/tqdm_1679561871187/work
traitlets @ file:///C:/ci_311/traitlets_1676423290727/work
truststore @ file:///C:/b/abs_55z7b3r045/croot/truststore_1695245455435/work
Twisted @ file:///C:/b/abs_e7yqd811in/croot/twisted_1708702883769/work
twisted-iocpsupport @ file:///C:/ci_311/twisted-iocpsupport_1676447612160/work
typing_extensions==4.12.2
tzdata @ file:///croot/python-tzdata_1690578112552/work
tzlocal @ file:///C:/ci_311/tzlocal_1676439620276/work
uc-micro-py @ file:///C:/ci_311/uc-micro-py_1676457695423/work
ujson @ file:///C:/ci_311/ujson_1676434714224/work
umap-learn==0.5.5
Unidecode @ file:///tmp/build/80754af9/unidecode_1614712377438/work
urllib3 @ file:///C:/b/abs_0c3739ssy1/croot/urllib3_1707349314852/work
validators @ file:///tmp/build/80754af9/validators_1612286467315/work
w3lib @ file:///C:/b/abs_957begrwnl/croot/w3lib_1708640020760/work
watchdog @ file:///C:/ci_311/watchdog_1676457923624/work
wcwidth @ file:///Users/ktietz/demo/mc3/conda-bld/wcwidth_1629357192024/work
webencodings==0.5.1
websocket-client @ file:///C:/ci_311/websocket-client_1676426063281/work
Werkzeug @ file:///C:/b/abs_8578rs2ra_/croot/werkzeug_1679489759009/work
whatthepatch @ file:///C:/ci_311/whatthepatch_1678402578113/work
widgetsnbextension @ file:///C:/b/abs_derxhz1biv/croot/widgetsnbextension_1701273671518/work
win-inet-pton @ file:///C:/ci_311/win_inet_pton_1676425458225/work
wrapt @ file:///C:/ci_311/wrapt_1676432805090/work
xarray @ file:///C:/b/abs_5bkjiynp4e/croot/xarray_1689041498548/work
xlwings @ file:///C:/ci_311_rebuilds/xlwings_1679013429160/work
xyzservices @ file:///C:/ci_311/xyzservices_1676434829315/work
yapf @ file:///tmp/build/80754af9/yapf_1615749224965/work
yarl @ file:///C:/b/abs_8bxwdyhjvp/croot/yarl_1701105248152/work
zict @ file:///C:/b/abs_780gyydtbp/croot/zict_1695832899404/work
zipp @ file:///C:/b/abs_b0beoc27oa/croot/zipp_1704206963359/work
zope.interface @ file:///C:/ci_311/zope.interface_1676439868776/work
zstandard==0.19.0
```

For manuscripts utilizing custom algorithms or software that are central to the research but not yet described in published literature, software must be made available to editors and reviewers. We strongly encourage code deposition in a community repository (e.g. GitHub). See the Nature Portfolio guidelines for submitting code & software for further information.

Data

Policy information about availability of data

All manuscripts must include a data availability statement. This statement should provide the following information, where applicable:

- Accession codes, unique identifiers, or web links for publicly available datasets
- A description of any restrictions on data availability
- For clinical datasets or third party data, please ensure that the statement adheres to our policy

Original data (images, prompts, model outputs, ratings, summary statistics) are available in the supplementary information and extended data for full reproducibility, including direct hyperlinks to previously published cases which are all publicly accessible. In-house cases are characterized in further detail in Ferber et al, 2024, Autonomous Artificial Intelligence Agents for Clinical Decision Making in Oncology.

Research involving human participants, their data, or biological material

Policy information about studies with human participants or human data. See also policy information about sex, gender (identity/presentation), and sexual orientation and race, ethnicity and racism.

Reporting on sex and gender	Cases were distributed equally across sex (6f:4m) or chosen without knowledge of underlying sex (endoscopic and histopathological imaging).
Reporting on race, ethnicity, or other socially relevant groupings	provide a statement on whether written/signed informed consent was obtained by participants in the Reporting Summary and Methods. Please also provide information on participant compensation. Please clarify whether this is not applicable.
Population characteristics	See above
Recruitment	No participant compensation was provided as only anonymized images were used.
Ethics oversight	The overall analysis was approved by the Ethics commission of the Medical Faculty of the Technical University Dresden (BO-EK-444102022). Local data was obtained from Uniklinik RWTH Aachen under grant nr EK 028/19.

Note that full information on the approval of the study protocol must also be provided in the manuscript.

Field-specific reporting

Please select the one below that is the best fit for your research. If you are not sure, read the appropriate sections before making your selection.

☒ Life sciences ☐ Behavioural & social sciences ☐ Ecological, evolutionary & environmental sciences

For a reference copy of the document with all sections, see nature.com/documents/nr-reporting-summary-flat.pdf

Life sciences study design

All studies must disclose on these points even when the disclosure is negative.

Sample size	n=18 (n=3 per modality) 72 distinct prompts / model = 18 cases + 54 prompt injections = (1 contr.+ 3 PI) * 18 cases<br>72 dist. prompts/model * 3 iter. = 216 runs / model<br>216 runs * 3 models = 648<br><br>54 distinct prompts / model = 18 cases + 36 prompt injections = (1 contr.+ 2 PI) * 18 cases<br>54 dist. prompts/model * 3 iter. = 162 runs / Reka Core<br><br>= 810 observations
Data exclusions	Data generated via Gemini Pro 1.5 was excluded as it was purely missing data, as discussed in the manuscript. Exclusion criterion (< 50 % organ detection rate) was pre-specified.
Replication	All analysis have been successfully reproduced multiple times throughout the revision process
Randomization	Identical prompts were passed both to prompt injected and not-injected arms of the study, randomization was therefore not necessary.
Blinding	Outputs for injected vs not injected prompts were commenting on the output, in a blackbox-LLM study blinding was not possible.

Behavioural & social sciences study design

All studies must disclose on these points even when the disclosure is negative.

Study description	Quantitative, simulated study
Research sample	Exemplary samples from different oncological imaging data types
Sampling strategy	Random sampling
Data collection	After concept incl. 18 different example patients was established, a qualitative search for representative images both on our hospital servers and publicly available images fitting the description of e.g. "Liver tumor"<br>No participant consent was needed as data consisted of images only and is therefore anonymized/was obtained through external sources for which informed consent is a prerequisite for upload of information.
Timing	8-12.6.2024 ; 2-5.8.2024

Data exclusions	No exclusions of primary data
Non-participation	No drop outs
Randomization	NO randomization

Ecological, evolutionary & environmental sciences study design

All studies must disclose on these points even when the disclosure is negative.

Study description	*Briefly describe the study. For quantitative data include treatment factors and interactions, design structure (e.g. factorial, nested, hierarchical), nature and number of experimental units and replicates.*
Research sample	*Describe the research sample (e.g. a group of tagged Passer domesticus, all Stenocereus thurberi within Organ Pipe Cactus National Monument), and provide a rationale for the sample choice. When relevant, describe the organism taxa, source, sex, age range and any manipulations. State what population the sample is meant to represent when applicable. For studies involving existing datasets, describe the data and its source.*
Sampling strategy	*Note the sampling procedure. Describe the statistical methods that were used to predetermine sample size OR if no sample-size calculation was performed, describe how sample sizes were chosen and provide a rationale for why these sample sizes are sufficient.*
Data collection	*Describe the data collection procedure, including who recorded the data and how.*
Timing and spatial scale	*Indicate the start and stop dates of data collection, noting the frequency and periodicity of sampling and providing a rationale for these choices. If there is a gap between collection periods, state the dates for each sample cohort. Specify the spatial scale from which the data are taken*
Data exclusions	*If no data were excluded from the analyses, state so OR if data were excluded, describe the exclusions and the rationale behind them, indicating whether exclusion criteria were pre-established.*
Reproducibility	*Describe the measures taken to verify the reproducibility of experimental findings. For each experiment, note whether any attempts to repeat the experiment failed OR state that all attempts to repeat the experiment were successful.*
Randomization	*Describe how samples/organisms/participants were allocated into groups. If allocation was not random, describe how covariates were controlled. If this is not relevant to your study, explain why.*
Blinding	*Describe the extent of blinding used during data acquisition and analysis. If blinding was not possible, describe why OR explain why blinding was not relevant to your study.*

Did the study involve field work? ☐ Yes ☒ No

Reporting for specific materials, systems and methods

We require information from authors about some types of materials, experimental systems and methods used in many studies. Here, indicate whether each material, system or method listed is relevant to your study. If you are not sure if a list item applies to your research, read the appropriate section before selecting a response.

Materials & experimental systems

n/a	Involved in the study	
☐	☐	Antibodies
☐	☐	Eukaryotic cell lines
☐	☐	Palaeontology and archaeology
☐	☐	Animals and other organisms
☐	☐	Clinical data
☐	☐	Dual use research of concern
☐	☐	Plants

Methods

n/a	Involved in the study	
☐	☐	ChIP-seq
☐	☐	Flow cytometry
☐	☐	MRI-based neuroimaging

Antibodies

Antibodies used	*Describe all antibodies used in the study; as applicable, provide supplier name, catalog number, clone name, and lot number.*
Validation	*Describe the validation of each primary antibody for the species and application, noting any validation statements on the manufacturer's website, relevant citations, antibody profiles in online databases, or data provided in the manuscript.*

Eukaryotic cell lines

Policy information about cell lines and Sex and Gender in Research

Cell line source(s)	*State the source of each cell line used and the sex of all primary cell lines and cells derived from human participants or vertebrate models.*
Authentication	*Describe the authentication procedures for each cell line used OR declare that none of the cell lines used were authenticated.*
Mycoplasma contamination	*Confirm that all cell lines tested negative for mycoplasma contamination OR describe the results of the testing for mycoplasma contamination OR declare that the cell lines were not tested for mycoplasma contamination.*
Commonly misidentified lines (See ICLAC register)	*Name any commonly misidentified cell lines used in the study and provide a rationale for their use.*

Palaeontology and Archaeology

Specimen provenance	*Provide provenance information for specimens and describe permits that were obtained for the work (including the name of the issuing authority, the date of issue, and any identifying information). Permits should encompass collection and, where applicable, export.*
Specimen deposition	*Indicate where the specimens have been deposited to permit free access by other researchers.*
Dating methods	*If new dates are provided, describe how they were obtained (e.g. collection, storage, sample pretreatment and measurement), where they were obtained (i.e. lab name), the calibration program and the protocol for quality assurance OR state that no new dates are provided.*

☐ Tick this box to confirm that the raw and calibrated dates are available in the paper or in Supplementary Information.

Ethics oversight	*Identify the organization(s) that approved or provided guidance on the study protocol, OR state that no ethical approval or guidance was required and explain why not.*

Note that full information on the approval of the study protocol must also be provided in the manuscript.

Animals and other research organisms

Policy information about studies involving animals; ARRIVE guidelines recommended for reporting animal research, and Sex and Gender in Research

Laboratory animals	*For laboratory animals, report species, strain and age OR state that the study did not involve laboratory animals.*
Wild animals	*Provide details on animals observed in or captured in the field; report species and age where possible. Describe how animals were caught and transported and what happened to captive animals after the study (if killed, explain why and describe method; if released, say where and when) OR state that the study did not involve wild animals.*
Reporting on sex	*Indicate if findings apply to only one sex; describe whether sex was considered in study design, methods used for assigning sex. Provide data disaggregated for sex where this information has been collected in the source data as appropriate; provide overall numbers in this Reporting Summary. Please state if this information has not been collected. Report sex-based analyses where performed, justify reasons for lack of sex-based analysis.*
Field-collected samples	*For laboratory work with field-collected samples, describe all relevant parameters such as housing, maintenance, temperature, photoperiod and end-of-experiment protocol OR state that the study did not involve samples collected from the field.*
Ethics oversight	*Identify the organization(s) that approved or provided guidance on the study protocol, OR state that no ethical approval or guidance was required and explain why not.*

Note that full information on the approval of the study protocol must also be provided in the manuscript.

Clinical data

Policy information about clinical studies

All manuscripts should comply with the ICMJE guidelines for publication of clinical research and a completed CONSORT checklist must be included with all submissions.

Clinical trial registration	*Provide the trial registration number from ClinicalTrials.gov or an equivalent agency.*
Study protocol	*Note where the full trial protocol can be accessed OR if not available, explain why.*
Data collection	*Describe the settings and locales of data collection, noting the time periods of recruitment and data collection.*

Outcomes

Describe how you pre-defined primary and secondary outcome measures and how you assessed these measures.

Dual use research of concern

Policy information about dual use research of concern

Hazards

Could the accidental, deliberate or reckless misuse of agents or technologies generated in the work, or the application of information presented in the manuscript, pose a threat to:

No	Yes	
☐	☒	Public health
☐	☒	National security
☐	☐	Crops and/or livestock
☐	☐	Ecosystems
☐	☐	Any other significant area

Experiments of concern

Does the work involve any of these experiments of concern:

No	Yes	
☐	☐	Demonstrate how to render a vaccine ineffective
☐	☐	Confer resistance to therapeutically useful antibiotics or antiviral agents
☐	☐	Enhance the virulence of a pathogen or render a nonpathogen virulent
☐	☐	Increase transmissibility of a pathogen
☐	☐	Alter the host range of a pathogen
☐	☒	Enable evasion of diagnostic/detection modalities
☐	☐	Enable the weaponization of a biological agent or toxin
☐	☒	Any other potentially harmful combination of experiments and agents

Plants

Seed stocks

Report on the source of all seed stocks or other plant material used. If applicable, state the seed stock centre and catalogue number. If plant specimens were collected from the field, describe the collection location, date and sampling procedures.

Novel plant genotypes

Describe the methods by which all novel plant genotypes were produced. This includes those generated by transgenic approaches, gene editing, chemical/radiation-based mutagenesis and hybridization. For transgenic lines, describe the transformation method, the number of independent lines analyzed and the generation upon which experiments were performed. For gene-edited lines, describe the editor used, the endogenous sequence targeted for editing, the targeting guide RNA sequence (if applicable) and how the editor was applied.

Authentication

Describe any authentication procedures for each seed stock used or novel genotype generated. Describe any experiments used to assess the effect of a mutation and, where applicable, how potential secondary effects (e.g. second site T-DNA insertions, mosiacism, off-target gene editing) were examined.

ChIP-seq

Data deposition

☐ Confirm that both raw and final processed data have been deposited in a public database such as GEO.

☐ Confirm that you have deposited or provided access to graph files (e.g. BED files) for the called peaks.

Data access links
May remain private before publication.

For "Initial submission" or "Revised version" documents, provide reviewer access links. For your "Final submission" document, provide a link to the deposited data.

Files in database submission

Provide a list of all files available in the database submission.

Genome browser session
(e.g. UCSC)

Provide a link to an anonymized genome browser session for "Initial submission" and "Revised version" documents only, to enable peer review. Write "no longer applicable" for "Final submission" documents.

Methodology

Replicates	*Describe the experimental replicates, specifying number, type and replicate agreement.*
Sequencing depth	*Describe the sequencing depth for each experiment, providing the total number of reads, uniquely mapped reads, length of reads and whether they were paired- or single-end.*
Antibodies	*Describe the antibodies used for the ChIP-seq experiments; as applicable, provide supplier name, catalog number, clone name, and lot number.*
Peak calling parameters	*Specify the command line program and parameters used for read mapping and peak calling, including the ChIP, control and index files used.*
Data quality	*Describe the methods used to ensure data quality in full detail, including how many peaks are at FDR 5% and above 5-fold enrichment.*
Software	*Describe the software used to collect and analyze the ChIP-seq data. For custom code that has been deposited into a community repository, provide accession details.*

Flow Cytometry

Plots

Confirm that:

☐ The axis labels state the marker and fluorochrome used (e.g. CD4-FITC).

☐ The axis scales are clearly visible. Include numbers along axes only for bottom left plot of group (a 'group' is an analysis of identical markers).

☐ All plots are contour plots with outliers or pseudocolor plots.

☐ A numerical value for number of cells or percentage (with statistics) is provided.

Methodology

Sample preparation	*Describe the sample preparation, detailing the biological source of the cells and any tissue processing steps used.*
Instrument	*Identify the instrument used for data collection, specifying make and model number.*
Software	*Describe the software used to collect and analyze the flow cytometry data. For custom code that has been deposited into a community repository, provide accession details.*
Cell population abundance	*Describe the abundance of the relevant cell populations within post-sort fractions, providing details on the purity of the samples and how it was determined.*
Gating strategy	*Describe the gating strategy used for all relevant experiments, specifying the preliminary FSC/SSC gates of the starting cell population, indicating where boundaries between "positive" and "negative" staining cell populations are defined.*

☐ Tick this box to confirm that a figure exemplifying the gating strategy is provided in the Supplementary Information.

Magnetic resonance imaging

Experimental design

Design type	*Indicate task or resting state; event-related or block design.*
Design specifications	*Specify the number of blocks, trials or experimental units per session and/or subject, and specify the length of each trial or block (if trials are blocked) and interval between trials.*
Behavioral performance measures	*State number and/or type of variables recorded (e.g. correct button press, response time) and what statistics were used to establish that the subjects were performing the task as expected (e.g. mean, range, and/or standard deviation across subjects).*

Acquisition

Imaging type(s) | *Specify: functional, structural, diffusion, perfusion.*

Field strength | *Specify in Tesla*

Sequence & imaging parameters | *Specify the pulse sequence type (gradient echo, spin echo, etc.), imaging type (EPI, spiral, etc.), field of view, matrix size, slice thickness, orientation and TE/TR/flip angle.*

Area of acquisition | *State whether a whole brain scan was used OR define the area of acquisition, describing how the region was determined.*

Diffusion MRI ☐ Used ☐ Not used

Preprocessing

Preprocessing software | *Provide detail on software version and revision number and on specific parameters (model/functions, brain extraction, segmentation, smoothing kernel size, etc.).*

Normalization | *If data were normalized/standardized, describe the approach(es): specify linear or non-linear and define image types used for transformation OR indicate that data were not normalized and explain rationale for lack of normalization.*

Normalization template | *Describe the template used for normalization/transformation, specifying subject space or group standardized space (e.g. original Talairach, MNI305, ICBM152) OR indicate that the data were not normalized.*

Noise and artifact removal | *Describe your procedure(s) for artifact and structured noise removal, specifying motion parameters, tissue signals and physiological signals (heart rate, respiration).*

Volume censoring | *Define your software and/or method and criteria for volume censoring, and state the extent of such censoring.*

Statistical modeling & inference

Model type and settings | *Specify type (mass univariate, multivariate, RSA, predictive, etc.) and describe essential details of the model at the first and second levels (e.g. fixed, random or mixed effects; drift or auto-correlation).*

Effect(s) tested | *Define precise effect in terms of the task or stimulus conditions instead of psychological concepts and indicate whether ANOVA or factorial designs were used.*

Specify type of analysis: ☐ Whole brain ☐ ROI-based ☐ Both

Statistic type for inference | *Specify voxel-wise or cluster-wise and report all relevant parameters for cluster-wise methods.*

(See Eklund et al. 2016)

Correction | *Describe the type of correction and how it is obtained for multiple comparisons (e.g. FWE, FDR, permutation or Monte Carlo).*

Models & analysis

n/a	Involved in the study
☐	☐ Functional and/or effective connectivity
☐	☐ Graph analysis
☐	☐ Multivariate modeling or predictive analysis

Functional and/or effective connectivity | *Report the measures of dependence used and the model details (e.g. Pearson correlation, partial correlation, mutual information).*

Graph analysis | *Report the dependent variable and connectivity measure, specifying weighted graph or binarized graph, subject- or group-level, and the global and/or node summaries used (e.g. clustering coefficient, efficiency, etc.).*

Multivariate modeling and predictive analysis | *Specify independent variables, features extraction and dimension reduction, model, training and evaluation metrics.*
